# 

**Published:** 1889-11

**Authors:** 


					﻿r '	i
188©.
OLD SERIES
Vol. xxv
_	<? 1855 0-
NEW SF.RieS
h Vol. VI.-No. 9,.
|fe
Wedigal^uS
JmiRNA^B
Wbj
& W. F. WESTMORELAND, M.D.
H. V. M. MILLER, M.D., LL.D.
VIRGIL 0. HARDON, M.D.
F. W. McRAE, M.D., Man. & Pro.
IT	O 11#	__^«rc*
MjBUSHED By JAMES P.HARRISON&Col
VP______________«flT LANTA, GA.
SUBSCRIPTION E 12.00 A YEAR
				

